# Proceedings of Southern Dental Association

**Published:** 1881-11

**Authors:** 


### ARTICLE V.

### *Southern Dental Association.—Annual Meeting.*

[Continued from last Number.]

(CONJOINTLY WITH THE NORTH CAROLINA STATE DENTAL ASSOCIATION.)

THIRD DAY, *Morning Session.*

Baltimore, Maryland, was selected as the place for holding the next annual meeting, and the second Tuesday in August, 1882, selected as the day.

The secretary read a paper from Dr. H. J. Kimball, of Atlanta, Georgia, inviting the Association to meet in that city during the coming cotton exposition.

The following report was read:

Your committee on appliances, after careful examination of the Portable Dental Chair, presented by Dr. A. W. Ross, of South Carolina, would say that it is the best thing of the kind ever offered to the profession, and when properly manufactured, will no doubt become a necessity to the itinerant Dentist.

H. M. GRANT,
I. SIMPSON,
GEO. W. WINKLER.

A paper from Dr. T. B. Allen, of Eufola, Alabama, on Dental Therapeutics was read.

Dr. Hodgkin spoke upon the recent introduction of Eucalyptus oil as a substitute for carbolic acid in the treatment of dead teeth. Used in conjunction with iodoform it seemed quite a valuable agent to our therapy.

Dr. Grant confirmed this statement.

Dr. Winkler had found Pond's extract and lead water a useful combination; but the best gum wash he had met with was,

℞.—Acid Carbolic, glts, xxxv.

Glycerine, - - ʒ i.

Aqua, - ℥ iv.

It relieves the pain felt after extracting teeth speedily, and was useful in abscesses. Bromide of potash was also useful, and quinine especially so if given in large doses.

Dr. Walker endorsed Pond's extract as useful, and Dr. Hunter tincture of myrrh, which he thought he had seen do good in cases of Riggs' disease.

Dr. Winkler again argued the claims of quinine. It made a most excellent local application in solution, three grains to an ounce of water, in gonorrhœa.

Dr. Hunter thought too much importance could not be given to quinine as a medical agent in our malarious climates. He used it in large doses with good effect in cases of alveolar abscess.

Dr. Chisholm and others spoke of the usefulness of the same drug.

Dental Hygiene was called.

Dr. Walker, of New Orleans, read the following paper:

The marked neglect which this study has received is a subject of common comment; so much so indeed that the assertion has been made and widely repeated that no one has come forward to tell us how to prevent the decay of teeth. That is the business of Hygiene; it is prophylactic.

I only hope I may be able to shed a little light upon this subject. The wide field and the limited time preclude the possibility of much elaboration, and renders brevity necessary.

My paper is a summary of experience rather than an argument.

First, *why* and *how* do teeth decay?

A series of observations extending through a dental practice of over thirty years—more than half of which has been spent in a locality peculiarly favorable for studying this subject—has resulted in the conviction that the chief, and perhaps primary cause of defective teeth, is imperfect nutrition.

Dr. Norman W. Kingsley, in a recent paper, after enumerating a long list of special causes of decay, and denying to some extent, the effective force of each one, gives a final answer to the question in these words: "teeth decay, primarily, because the nutrition of these organic structures being obstructed, retrograde metamorphosis ensues."

This condition he attributes to what he calls *civilization*, but to this same civilization he naturally, and very justly looks for the perfect remedy.

The obstruction of nutrition he attributes largely to the nervous condition produced in civilized communities by overwork, and taxing the brain and nervous system at the expense of the general welfare of the body.

While this is undoubtedly true to a considerable degree, observation has convinced me that the difficulty is owing in a much greater degree to the inefficiency of proper alimentary substance.

I think Dr. Kingsley rightly attributes our defective teeth to *civilization*, and I think also that the hope that he expresses is well founded; that a still higher and more perfect civilization will restore us to a normal and healthy condition.

As to *how* teeth decay, it is manifest a process of vital chemistry.

As to *why* they decay, I think we may safely say, first, because of imperfect constitutional character; secondly to being surrounded with conditions which tend to disorganize and destroy, and are able to do so the more readily owing to this imperfect constitutional character.

I think it manifest to all close observers, that during the entire extent of life, nutrient action is carried on throughout the substance of the tooth, and that "retrograde metamorphosis," so called, occurs when this nutrient action removes the solid substance of the tooth, but fails to supply its place with new material; not so much because of *obstruction* which would interfere with the removal of the lime-salts as well, as from the failure to supply the proper nutrient element to replace the debris which has been removed.

To illustrate—immigrants from the mountainous regions of Europe or America, whose advantages of plain, substantial, nourishing food; good, pure well or spring water; fresh pure air, and abundant exercise, have furnished them with teeth, admirable in beauty of form and solidity of structure—changing locality, and habits, and diet—using superfine flour bread and pastry; drinking cistern water, and breathing impure air, are subject to what is called "retrograde metamorphosis," in that the substance which rendered their teeth hard and firm is removed, while the supply is so deficient that these beautiful teeth become softened throughout; they have become deficient in mineral elements; the connective tissue becomes prominent; they become super-sensitive, and are readily acted upon by the acids which are used, whether as condiments, or acids from fruits, or from over-cooked preserves, or from sweets turning sour, or more especially from the great and destructive enemy of teeth—starchy, pasty food, left lying about them.

"Retrograde metamorphosis" does not properly express the change in tooth, meaning a change in form, while what really occurs is a change of substance.

Our emigrant having suffered this constitutional deterioration, begets children who not only inherit his physical demoralization, but who also, in addition to this, inherit the habits and conditions which produced it.

What may be termed *constitutional habit* is also an important factor in the deterioration of teeth.

Nutrient organs that are accustomed to an abundant supply, acquire a habit of depending upon that abundance which renders them incapable of extracting the full necessary amount of nutrient elements out of a more meagre supply, from which the nutrient organs of an individual long accustomed to this meagre supply, would be able to extract the amount necessary for perfect nutrition.

Thus the descendants of emigrants, who on settling in the Mississippi delta, have suffered great deterioration of their teeth, in from four to five generations, become adapted to the new conditions, and out of the meagre supply of nutrient elements furnished are unable to produce good teeth.

A knowledge of the cause of our troubles points naturally to the remedy, which is—first, to supply the proper nutrient elements, in diet and drink to render the teeth hard and strong, and as far as possible, impervious to bad surrounding conditions; second, to educate our patients into habits of such cleanliness as will remove the pernicious surroundings which will attack even good teeth, and are so very destructive to weak ones.

The most efficient remedy which I have found, for connecting these unfortunate conditions—beyond furnishing proper food—is an aqueous solution of carbonate of lime.

With the proper administration of lime-water, and such improvement in food as can be easily made, it is possible not only to prevent deterioration, but actually to improve the stock, overcome bad heredity, and give the new generation better teeth than those of our predecessors.

Another branch of the subject may properly claim our attention. A proper consideration of hygienic conditions will, I think, induce us to change very largely some of the modes of operating, adopted heretofore, with a view to preserving teeth.

Creative wisdom has placed the last tooth in the lower jaw so as to be supported by the angle of the jaw, and so form the firm base of an arch, rendered substantial by the contact of each tooth with its fellow, at or near the grinding surface, with the best possible conditions for cleanliness; the triangular spaces between the teeth enabling the gum to grow well-around their necks, and affording opportunity for the passage of washing fluids, thus naturally removing any acids or deleterious substance.

The teeth of the upper jaw are so placed as to take their form, and be guided in their position by those of the lower jaw; the whole forming one of the most beautiful and practical illustrations of Divine wisdom to be found in Anatomy. Unfortunately, many of our predecessors, failing to comprehend the practical beauty of this arrangement, deeming themselves capable of improving upon the plan of Divine wisdom, have introduced a real "retrograde metamorphosis," or change of form; and instead of leaving the teeth to support each other, according to the Divine plan, their effort has been to have each tooth stand independent of each other.

This has however, proved an utter failure because nature, ever jealous of interference in her well-planned arrangements, makes an effort to recover the least support furnished by each tooth to its fellow. The result is that the dental arch is contracted, and infringes upon the tongue; interferes with its free play in perfect articulation, while mastication is rendered imperfect from the diminution of grinding surface. Or, if the teeth fail to come together again, the food is crowded into the spaces left, producing irritation of the gum, and denudation of the teeth.

When they do come together, the point of contact, instead of being a well-rounded and well-protected point, is a flat surface, with the protecting enamel already removed; or they touch at the shoulders, near the gum, around which food packs, and produces decay; and altogether the condition of that patient's mouth is far worse than before it was touched.

Therefore I say, that one of the greatest abominations, which a proper consideration of hygienic conditions should cause us to abandon and abolish forever, is that hideous thing known as a *separating file.*

Another abomination against which we should set our faces most earnestly, is the common and almost universa use of *stiff tooth-brushes.* In probably nine-tenths of the drug-stores of the country, it is almost an impossibility to get a suitable tooth-brush. People have been taught to brush their teeth cross-ways, with hard, stiff brushes, the friction of which, while it renders the gums at first unnaturally hard, finally results in driving it away from its proper position, not only exposing the teeth beyond the enamel, but absolutely removes the enamel and in some cases a large portion of the tooth. I have in my possession a cast of the teeth of a patient, of which a fourth to a half has been absolutely brushed away. Notches and grooves worn into the teeth, are not unusual occurrences.

At the same time that this immense amount of labor and wear is put upon teeth, the most important part of their cleansing is left undone; that is, the thorough removal of the food from between the teeth by the use of the tooth-pick or thread.

We should use every means in our power to abolish the use of large, stiff, hard, brushes; instructing our patients to procure, or furnishing them with small, convenient, soft, brushes, which can be brought to bear upon all exposed surfaces of the teeth; and then teach them to use them lengthwise upon these organs so as to draw the gums on to the teeth, and maintain its normal and healthy condition.

We should also insist upon the thorough use of the quill tooth-pick, or loose thread of flax or silk, to thoroughly cleanse those most important places, the approximal surfaces; at the same time endeavoring to dissipate the erroneous impression which unfortunately prevails, especially among ladies, that there is something indelicate or improper in the use of the tooth pick.

They should be taught that the sight of teeth packed with particles of food is far more indelicate and revolting than the proper use of the tooth-pick.

Whenever we succeed in bringing about that "higher civilization" toward which Dr. Kingsley, in common with all other intelligent men, is looking, our patients will use such food as is best calculated to develope and maintain the normal and healthy condition of the teeth, as well as to maintain general good health.

We should cease to mutilate these beautiful organs, or attempt to improve upon the Divine plan in their construction, but shall maintain them in full and perfect configuration, and perfect usefulness; and the eye will no longer be offended by the display of the decayed and fragmentary contents of an oral cavity, where all should be sweetness, purity, and grace.

Prof. Hodgkin.—That is an interesting and excellent paper, almost as much so as if it were all true. We have not time now to discuss it, but if it can be laid over until our next annual meeting I think I can prove the incorrectness of Dr. Walker's opinions.

Prof. Winder.—I agree with Prof. Hodgkin, that all that is a very pretty theory, but it is not true. I hope Dr. Walker will look close into this matter and be prepared to discuss it at our next meeting.

The paper of Dr. Walker's was then laid over for discussion at the next annual meeting.

Dr. Lawrence, the Treasurer of the Association, made his annual report, showing a balance unprovided for of $190.

Arrangements were made for printing the proceedings of this session of the Association in THE AMERICAN JOURNAL OF DENTAL SCIENCE; Prof. Hodgkin agreeing to edit and prepare them for publication.

The executive committee was instructed to attend to all arrangements for clinics at the next session.

The newly elected officers were now installed, appropriate addresses being made by them on assuming office. The retiring President, Dr. Turner, tendered his thanks for the forbearance and courtesy of the Association toward him, and congratulated the members on their new and active presiding officer.

Votes of thanks were tendered the county authorities for the use of the court house building as the scene of the session of the Association, to the hotels and citizens of Asheville, and others.

On motion of Dr. Hunt a committee of three was appointed to revise the Constitution and By-laws of the Association, said committee to report at next annual meeting; the revised constitution to go into effect immediately after its adoption by the Association.

Prof. F. J. S. Gorgas, of Maryland, J. R. Walker, of Louisiana, and H. M. Grant, of Virginia, committee.

The Association adjourned to meet in Baltimore, Md., as per resolution.

---

